# Efficient utilization of Shuanghuanglian medicine residues by microbial transformation with flavonoid glycosides-hydrolyzing strains

**DOI:** 10.3389/fmicb.2025.1553399

**Published:** 2025-05-27

**Authors:** Xingwang Ma, Ying Chen, Lili Li, Tianwei Wang, Kunling Teng, Junchang Su, Lei Li, Liangliang Li, Heping Li, Wentao Diao, Guocan Chen, Jiwen Wang, Jin Zhong

**Affiliations:** ^1^State Key Laboratory of Microbial Resources, Institute of Microbiology, Chinese Academy of Sciences, Beijing, China; ^2^College of Life Sciences, University of Chinese Academy of Sciences, Beijing, China; ^3^Institute of Biology Co., Ltd., Henan Academy of Sciences, Zhengzhou, China; ^4^College of Veterinary Medicine, Henan Agricultural University, Zhengzhou, China

**Keywords:** Chinese medicine residue, flavonoid, *Lactiplantibacillus plantarum*, feed additive, intestinal microbiota

## Abstract

Shuanghuanglian, a traditional Chinese medicine, is well-known for its bioactive compounds, such as flavonoids, which offer significant health benefits. However, the production of Shuanghuanlian generates substantial pharmaceutical residues, which are often discarded as waste, posing significant environmental and economic challenges. To date, research on repurposing these medicine residues has been limited. This study utilized beneficial microbes to efficiently extract and utilize the residual bioactive compounds. Notably, a newly isolated *Lactiplantibacillus plantarum* strain LLB exhibited remarkable efficiency in converting flavonoid glycosides (e.g., phillyrin and luteoloside) into their corresponding aglycones. When combined synergistically with *Bacillus subtilis* and *Saccharomyces cerevisiae*, strain LLB maintained robust flavonoid glycoside conversion while enhancing lactobacilli viability in the fermented medicine residues. As a feed additive for broiler chickens, the fermented residue not only boosted antioxidant (superoxide dismutase) and anti-inflammatory (IL-10) markers, but also preserved growth performance and meat quality. Furthermore, the fermented residue modulated the gut microbiome, increasing *Rikenella* while reducing *Elusimicrobiota* and *Parabacteroides* abundances. Our findings demonstrate that microbial transformation of Shuanghuanglian residues offers a sustainable strategy for waste valorization and a novel feed additive for enhancing animal health.

## 1 Introduction

Shuanghuanglian (SHL), a traditional Chinese medicine, is formulated from extracts of three medicinal herbs: *Scutellaria baicalensis*, *Forsythia suspensa*, and *Lonicera japonica* (Peng et al., 2021). SHL exhibits diverse pharmacological activities, including antibacterial properties and immunomodulation (Li R. et al., 2022). Additionally, SHL can inhibit the 3-chymotrypsin-like protease (3CLpro), conferring antiviral activity against coronaviruses, a capability that has heightened its demand since the COVID-19 outbreak in 2019 (Su et al., 2020). Ethanol precipitation is the primary method for extracting the active ingredients of SHL preparation from the herbs (Gao Y. et al., 2022). However, the extraction method is inefficient, yielding a large number of medicinal residues. Typically, these residues are discarded in industrial processes, leading to serious environmental pollution and resource wastage (Huang C. et al., 2021). Notably, SHL residues retain numerous active substances, such as flavonoids (e.g., phillyrin and baicalin) and chlorogenic acid. These bioactive ingredients have been shown to promote animal growth and alleviate diarrhea (Kuralkar and Kuralkar, 2021; Sun et al., 2023). SHL residues also contain nutrients, including crude proteins, vitamins, and polysaccharides (Lu and Li, 2021). Therefore, SHL residue hold potential as a feed additive in animal production.

Fermented feeds have many advantages over unfermented feeds and have been widely used in animal husbandry (Sugiharto and Ranjitkar, 2019). The residues of traditional Chinese medicine are enriched through fermentation (Sun et al., 2023). The microbial glycosidases are capable of hydrolyzing the glycosidic moieties of flavonoids present in the residues and convert them into their respective aglycones (Feng et al., 2019). Aglycones, as opposed to their glycoside precursors, are generally recognized for enhanced intestinal absorption and believed to be more efficiently utilized (Xu et al., 2017).

To ensure the safety of fermented medicinal residues for use as feed additives, the selection of fermenting microorganisms is critical. Lactic acid bacteria, particularly lactobacilli, synthesize various glycosidases, such as α-rhamnosidases and β-glucosidases, effectively transforming active substances in medicine residues (Ferreira-Lazarte et al., 2021; Rokni et al., 2021). As prominent probiotics, lactobacilli exhibit activity against pathogenic bacteria, thereby promoting animal health (Azad et al., 2018). Beyond lactobacilli, *Bacillus subtilis* and *Saccharomyces cerevisiae* also synthesize various glycosidases (Kuranda and Robbins, 1987) and are commonly used in feed fermentation processes. *B. subtilis* is capable of producing substantial quantities of antibacterial substances, including lipopeptides and bacteriocins, that prevent the colonization of pathogenic bacteria in animal gastrointestinal tract (Caulier et al., 2019). Moreover, *S. cerevisiae* enhances the protein content and palatability of fermented feeds, augmenting their nutritional value (Guo et al., 2021). Overall, the application of these microorganisms not only enhances the bioactivity of medicinal residues but also enriches the nutritional profile of fermented feeds, offering a viable strategy for animal nutrition and health.

Broiler chickens have a global annual production of approximately 92 million metric tons (Crippen et al., 2019), with China ranking as the second-largest producer worldwide (Zong et al., 2022). With escalating global demand for broiler chickens, consumers are increasingly focused on the safety, palatability, and nutritional content of chicken meat. Pathogenic bacteria, such as *Salmonella* (*Salm*) *enterica*, *Escherichia coli*, and *Staphylococcus* (*Stap*) *aureus*, not only infect broiler chickens, resulting in diarrhea and mortality, but also pose a risk of foodborne illness in humans through contaminated meat and eggs (Xu et al., 2021). While antibiotics are commonly employed in conventional broiler farming to curb pathogenic bacteria, their excessive use fosters antibiotic resistance and can result in harmful meat residues, endangering human health (Huang P. et al., 2021). Research indicates that flavonoids present in SHL residues effectively inhibit pathogenic bacteria (Xu et al., 2019; Chen et al., 2022). In addition, the incorporation of beneficial microbes, such as *Lactiplantibacillus* (*Lpb*) *plantarum* and *B. subtilis*, during the fermentation of SHL residues can regulate gut microbial homeostasis and significantly enhance the growth performance of broiler chickens.

Recent studies have explored the potential of traditional Chinese medicine (TCM) as a feed additive, offering an alternative to antibiotics in livestock and poultry breeding (Dittoe et al., 2022; Gao J. et al., 2022). Nevertheless, the fermentation of TCM residues and their applications in the breeding industry remain understudied areas. This study employed functional strains of *Lpb. plantarum*, *B. subtilis*, and *S. cerevisiae* to ferment SHL medicine residues, converting flavonoid glycosides into bioavailable aglycones for enhanced animal absorption. Subsequently, the fermented residue was incorporated as a feed additive in broiler chicken diets, and its impact on the intestinal microbiota and immune systems was evaluated. This research presents an effective strategy for repurposing TCM residues, potentially enhancing animal health through the application of fermented SHL residues as a feed additive.

## 2 Materials and methods

### 2.1 Isolation and identification of lactobacilli from SHL medicine residues for fermentation

To isolate and identify lactobacilli from SHL medicine residues, the De Man Rogosa Sharpe (MRS) medium was utilized (De Man et al., 1960). Amphotericin was added to the MRS medium at a final concentration of 20 μg/mL to inhibit fungal growth within the medicine residues. SHL medicine residues were appropriately diluted with sterile water and then plated onto MRS plates. After 48 h of incubation at 37°C, colonies were selected and identified through 16S rRNA gene sequencing. To confirm the species, genome sequencing was employed and the average nucleotide identity was calculated (Yoon et al., 2017) between the isolated strain and the reference strain.

### 2.2 Preparation of the flavonoid standard curve

Reversed-phase high-performance liquid chromatography (RP-HPLC) was used to analyze the flavonoid standard (Li R. et al., 2022). Chromatographic separation was carried out using a Supersil Phenyl column (5 μm, 4.6 mm × 250 mm). The mobile phase consisted of 0.1% trifluoroacetic acid in water (solvent A) and acetonitrile (solvent B). With a flow rate of 1.0 mL/min, the gradient elution program was as follows: 5% B at 0–5 min; 20–60% B at 5–45 min; 95% B at 45–50 min; and 5% B at 50–63 min. The injection volume was 10 μL.

### 2.3 Detection of the flavonoid glycoside-hydrolyzing abilities of strains used in SHL medicine residue fermentation

The abilities of *B. subtilis* C5B1 (C5B1), *S. cerevisiae* Lo-1 (Lo-1), and *Lpb. plantarum* LLB (LLB) to hydrolyze flavonoid glycosides were determined. Specifically, the strains were cultured in Luria-Bertani (LB) medium, yeast extract peptone dextrose (YPD) medium, or MRS medium. After these strains were grown to stationary phase, they were inoculated into fresh medium to achieve a final OD_600_ of 0.05. Subsequently, flavonoid glycosides were added at the following final concentrations: 8 μg/mL luteoloside, 20 μg/mL baicalin, and 40 μg/mL phillyrin. After a 24-h incubation period, the glycosides and their corresponding aglycones were analyzed using RP-HPLC as described above. The ratios of unhydrolyzed flavonoid glycosides were determined by dividing the residual content of each glycoside by its initial content. Similarly, the ratios of released aglycones were calculated by dividing the amount of aglycone produced by the initial amount of the corresponding glycoside.

### 2.4 Analysis of the antimicrobial activities of the fermenting strains

The antimicrobial activity was determined as previously described with some minor modifications (Li et al., 2023). *B. subtilis* C5B1, *S. cerevisiae* Lo-1, and *Lpb. plantarum* LLB were grown to the stationary phase. Subsequently, the cells were removed by centrifugation, and the supernatants were collected and filtered through a 0.22 μm sterile filter membrane. To assess the antimicrobial activity of the aforementioned strains, pathogens including *Candida albicans* SC5314, *Escherichia coli* DH5α, *Salmonella enterica* CGMCC 1.1859, and *Streptococcus equi* CGMCC 1.10838 were used as indicator organisms. These indicator bacteria were grown to the stationary phase and resuspended in fresh medium to achieve a concentration of 1 × 10^6^ CFU/mL. Then, 20% of the fermentation supernatants were added to the suspensions of the indicator bacteria. After 24 h, the OD_600_ values of the cultures were measured to determine the antimicrobial activities.

### 2.5 Preparation of fermented SHL medicine residues

The SHL medicine residues were mixed with 45% (w/w) sterile water and 5% (w/w) molasses for fermentation. Then the mixed medicine residues were separately inoculated with different microbes as described below (Table 1): no microbe (CK), C5B1, Lo-1, LLB, C5B1 and LLB (CL), Lo-1 and LLB (LoL), C5B1, Lo-1, and LLB (ALL). All groups underwent aerobic fermentation at 30°C for 2 days, followed by anaerobic fermentation for 5 days at 30°C. Note that *B. subtilis* and *S. cerevisiae* were added on day 0, while *Lpb. plantarum* was added after 2 days of aerobic fermentation.

**Table 1:** The fermentation process of SHL medicine residues.

Group	Strains	
	Aerobic fermentation (2 days)	Anaerobic fermentation (5 days)
CK	No exogenous microbes	–
C5B1	*B. subtilis* C5B1	–
Lo-1	*S. cerevisiae* Lo-1	–
LLB	–	*Lpb. plantarum* LLB
CL	*B. subtilis* C5B1	*Lpb. plantarum* LLB
LoL	*S. cerevisiae* Lo-1	*Lpb. plantarum* LLB
ALL	*B. subtilis* C5B1 and *S. cerevisiae* Lo-1	*Lpb. plantarum* LLB

–, no strain was added.

### 2.6 Analysis of microbial population and pH in the fermented medicine residues

The counting method for culturable microbes followed previous descriptions (Su et al., 2019) with some modifications. One gram of the fermented medicine residue was dissolved in 900 μL of sterile water and subjected to a 10-fold serial dilution. A total of 100 μL from each corresponding dilution was plated on the agar media. The numbers of colonies on MRS, YPD, and LB agar plates corresponding to the populations of lactobacilli, yeast, and *Bacillus* species, respectively, were manually counted. To determine the pH of the fermented medicine residues, 10 grams of each sample was dissolved in 90 mL of sterile water. The mixture was shaken at room temperature for 30 min, after which the pH value of the supernatant was measured using a pH meter.

### 2.7 Measurement of flavonoids from SHL medicine residues

Two grams of the medicine residue was resuspended in 20 mL of 70% methanol and were sonicated for 30 min to extract the flavonoids. After sonication, the supernatants were collected by centrifugation and filtered through a 0.22 μm sterile filter membrane. The filtered solutions were then freeze-dried using a vacuum freeze dryer and redissolved in 5 mL of 20% acetonitrile. The concentrations of flavonoids were examined as described above. The antimicrobial activities of the extracted substances were determined as mentioned previously.

### 2.8 Feeding experimental design

A total of 180 forty-day-old female Huangma chickens were selected and randomly divided into six groups with 30 chickens per group. The experimental room had an area of approximately 100 m^2^. The chickens were housed in cages, with two chickens per cage, arranged in three tiers. Throughout the experiment, each group received a specific diet corresponding to their respective treatments. The diets were formulated using standard commercial corn and soybean meal (the basal diet) and adjusted for each treatment group. The assigned groups were as follows (Table 2): basal diet (BD, group A), 95% BD + 5% unfermented medicine residues (group B), 95% BD + 5% CK (group C), 97.5% BD + 2.5% ALL (group D), 95% BD + 5% ALL (group E), and BD + 20 mg/kg aureomycin (group F). All chickens were raised under uniform management and environmental conditions. Water and feeds were provided *ad libitum*, and six chickens from each group were randomly selected and weighed weekly. The total duration of the trial was 70 days. At the end of the experiment, six chickens from each group were randomly selected to analyze the outcomes of the treatments. Blood samples from the jugular veins were collected in sterile tubes to separate the sera, which were stored at −80°C until further use. In addition, the cecal contents were collected to analyze the composition of the intestinal microbiota.

**Table 2:** The feeding experiment design.

Treatments	Feeds
Group A	Basal diet (BD)
Group B	95% BD + 5% unfermented medicine residues
Group C	95% BD + 5% CK
Group D	97.5% BD + 2.5% ALL
Group E	95% BD + 5% ALL
Group F	BD + 20 mg/kg aureomycin

### 2.9 Determination of the serum cytokines, antibodies and antioxidant activities

The levels of serum cytokines [IL-6 (Mreda, M130205), IL-10 (Mreda, M130197), and IFN-γ (G-CLONE, SEK-030021435)] and antibodies [IgA (Mreda, M054363), IgM (Mreda, M054363), and IgG (Mreda, M054381)], as well as the antioxidant activities indicated by superoxide dismutase (SOD) (Mreda, M130212), catalase (CAT) (Mreda, M149531), and total antioxidant capacity (T-AOC) (Jingkang, JLC10767), were examined. All of these factors were measured using commercially available enzyme-linked immunosorbent assay (ELISA) kits.

### 2.10 DNA extraction and 16S rRNA sequencing

Hundred milligrams of cecal content from each selected chicken was used to extract genomic DNA using the FastDNA SPIN Kit for Feces (MP-Biomedicals, United States). The V3-V4 regions of the 16s rRNA genes were amplified with 338F (5′-ACTCCTACGGGAGGCAGCAG-3′) and 806R (5′-GGACTACHVGGGTWTCTAAT-3′) and the products were used for high-throughput sequencing on an Illumina PE250 platform. The raw sequencing reads were deposited in National Microbiology Data Center with the accession number NMDC10018890.

### 2.11 Statistical analyses

Prior to one-way analysis of variance (ANOVA) and *t*-tests, the normality of data distributions was confirmed using Shapiro–Wilk tests, and homogeneity of variance was verified via Levene’s test. Depending on the nature of the comparison, either a two-tailed Student’s *t*-test or an ANOVA was used. Statistical significance was determined at a *p*-value threshold of less than 0.05.

## 3 Results and discussion

### 3.1 Screening and characterization of the strains used for fermenting the SHL medicine residues

**Figure 1:**
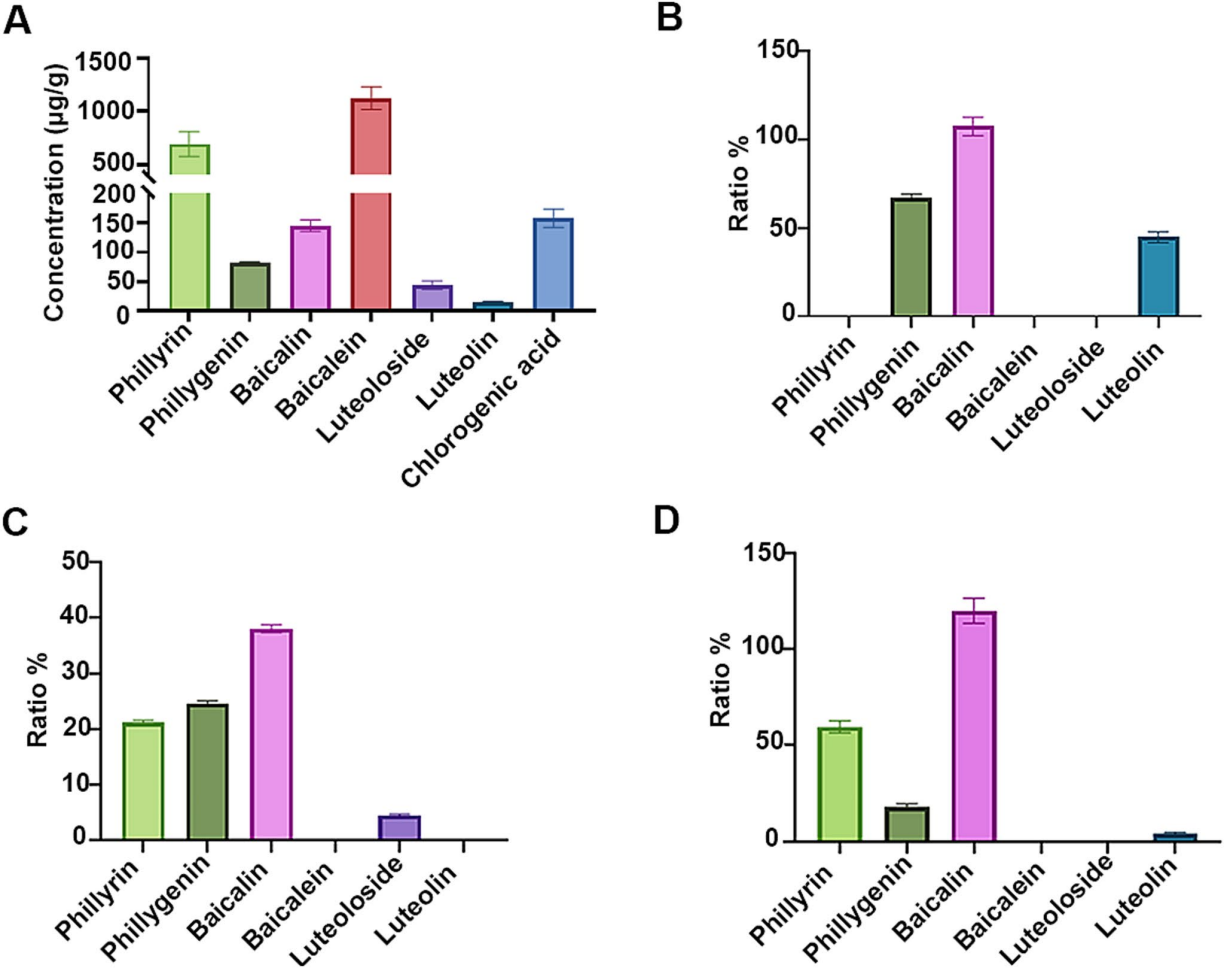
The flavonoid glycosides-hydrolyzing abilities of the fermenting strains. **(A)** Quantification of bioactive compounds in the unfermented Shuanghuanglian medicine residue. The ratios of the unhydrolyzed flavonoid glycosides and the released aglycones by *Lactiplantibacillus plantarum* LLB **(B)**, *Bacillus subtilis* C5B1 **(C)**, and *Saccharomyces cerevisiae* Lo-1 **(D)** in the presence of phillyrin, baicalin, and luteoloside, respectively.

As shown in Figure 1A, a significant number of flavonoid glycosides, notably phillyrin (approximately 690 μg/g) and baicalin (approximately 145 μg/g), remain in the SHL medicine residues. In pursuit of strains exhibiting robust flavonoid glycoside-hydrolyzing abilities and resistance to antibacterial substances present in the medicine residue, we isolated and characterized strains from the naturally fermented SHL residue. All colonies cultivated on MRS solid media were identified as *Lpb. plantarum*, indicating that *Lpb. plantarum* might be a dominant species within the microbial community of the fermented SHL residue. Among the isolated strains, strain LLB exhibited the highest growth rate in MRS medium and strong flavonoid glycoside-hydrolyzing activity (Figure 1B). The genome of strain LLB encodes two α-L-rhamnosidases, of which the coding genes are clustered like that in *Lpb. plantarum* NCC245 (Avila et al., 2009), and a β-D-glucosidase, enzymes pivotal for flavonoid glycoside hydrolysis. Beyond *Lpb. plantarum*, *B. subtilis* and *S. cerevisiae* also demonstrated the capacity to hydrolyze flavonoid glycosides (Wu and Chou, 2009; Schmidt et al., 2011). *B. subtilis* C5B1 produces entianin, a lanthipeptide with broad antibacterial activity against many foodborne pathogens, such as *Bacillus cereus* and *Listeria monocytogenes* (Liu et al., 2022). Unlike traditional antibiotics, lanthipeptides exhibit low susceptibility to drug resistance. Therefore, after fermentation by strain C5B1, SHL medicine residues should contain more antimicrobial substances, making them a potential alternative to antibiotics. *Saccharomyces cerevisiae* Lo-1 was isolated from fermented vinasses, which are rich in various metabolites similar to those found in fermented medicine residues. Thus, strain Lo-1 is expected to be suitable for growth in medicine residues and capable of transferring active substances. Therefore, we conducted a concurrent analysis of *B. subtilis* C5B1 and *S. cerevisiae* Lo-1 with strain LLB.

The ability of the fermenting strains to convert flavonoid glycosides in SHL medicine residue was assessed. As demonstrated in Figure 1B, *Lpb. plantarum* LLB completely consumed phillyrin and luteoloside, achieving hydrolysis ratios of 67.1 and 44.7% for the conversion to phillygenin and luteolin, respectively. The result highlighted the significant potential of *Lpb. plantarum* LLB for the utilization of active ingredients in medicine residues through the hydrolysis of flavonoid glycosides. Previous study had shown that *Lpb. plantarum* could hydrolyze flavonoid glycosides, but not those present in SHL residue (Avila et al., 2009; Beekwilder et al., 2009; Bai and Ganzle, 2015). *B. subtilis* C5B1 and *S. cerevisiae* Lo-1 also demonstrated the ability to convert phillyrin into phillygenin (Figures 1C,D), but their conversion efficiencies were inferior to that of *Lpb. plantarum* LLB. Additionally, *B. subtilis* C5B1 was observed to convert baicalin and luteoloside into molecules other than the corresponding aglycones. Likewise, *S. cerevisiae* Lo-1 have strong ability to transfer luteoloside, but the product only contained a little amount of luteolin. Except deglycosylation, many other types of microbial transformation on flavonoid glycosides are present (Cao et al., 2015). This suggested that *B. subtilis* C5B1 and *S. cerevisiae* Lo-1 might produce a range of enzymes beyond β-D-glucosidase, capable of diverse modifications to baicalin and luteoloside.

**Figure 2:**
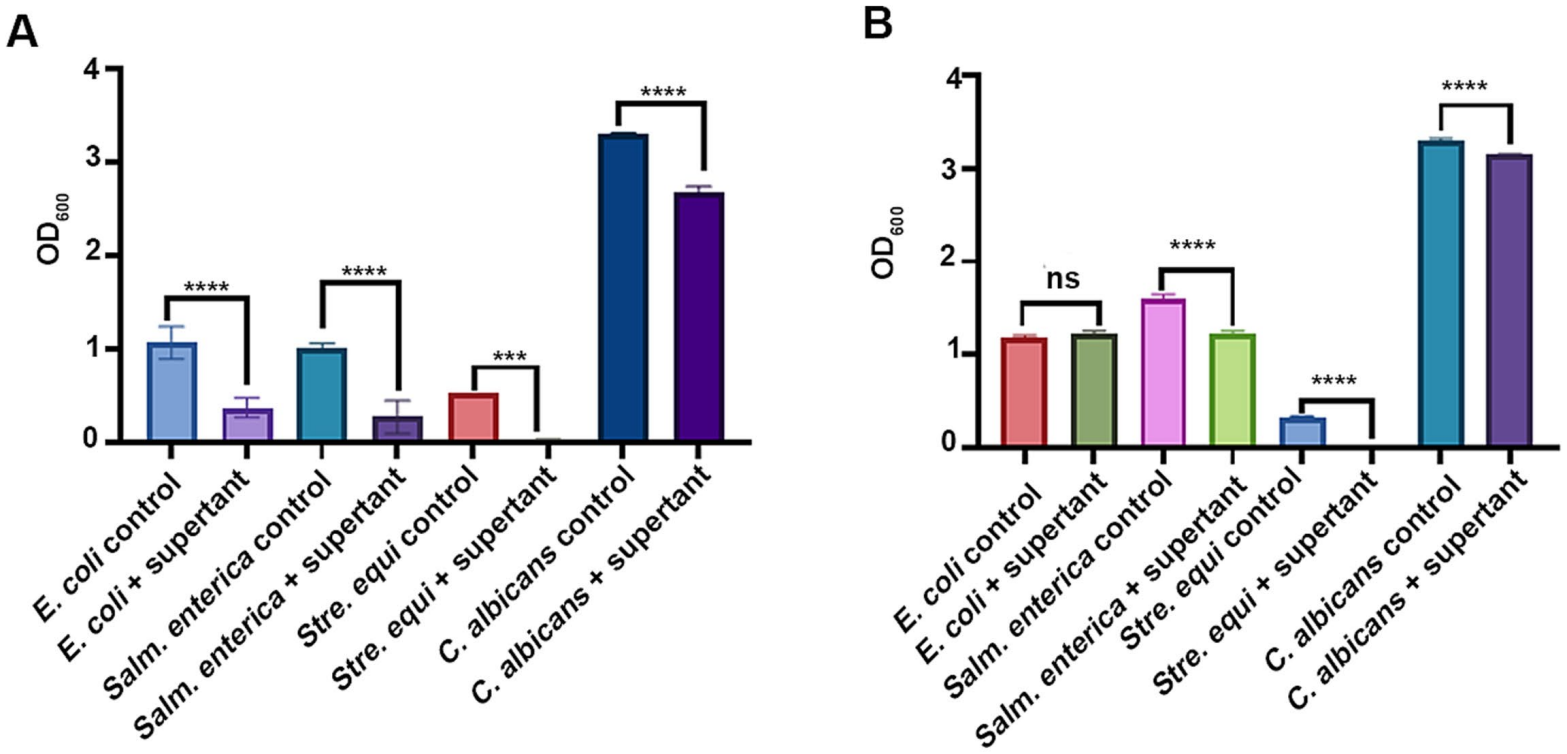
The antimicrobial abilities of *Lactiplantibacillus plantarum* LLB **(A)** and *Bacillus subtilis* C5B1 **(B)**. The indicators are common pathogenic microbes in chicken farming. ns, not significant; ***p < 0.001; ****p < 0.0001.

In addition, we evaluated the antimicrobial abilities of the fermenting strains, considering that *Lpb. plantarum* and *B. subtilis* are known producers of antimicrobial compounds, such as lactic acid and bacteriocins (Caulier et al., 2019; Rocchetti et al., 2021). Potential pathogenic microbes, including *E. coli*, *Salm. enterica, Stap. aureus*, *Streptococcus* (*Stre*) *equi*, and *Candida albicans*, served as indicators for assessing antimicrobial activity. As shown in Figure 2A, the fermentation supernatant from *Lpb. plantarum* LLB significantly inhibited the growth of all the indicators. In addition, *B. subtilis* C5B1 suppressed the growth of *Stre. equi* CGMCC 1.10838, *Salm. enterica* CGMCC 1.1859 and *C. albicans* SC5314 (Figure 2B).

### 3.2 Dynamic changes in the cultivable microbial population and pH value of the fermented medicine residues

**Figure 3:**
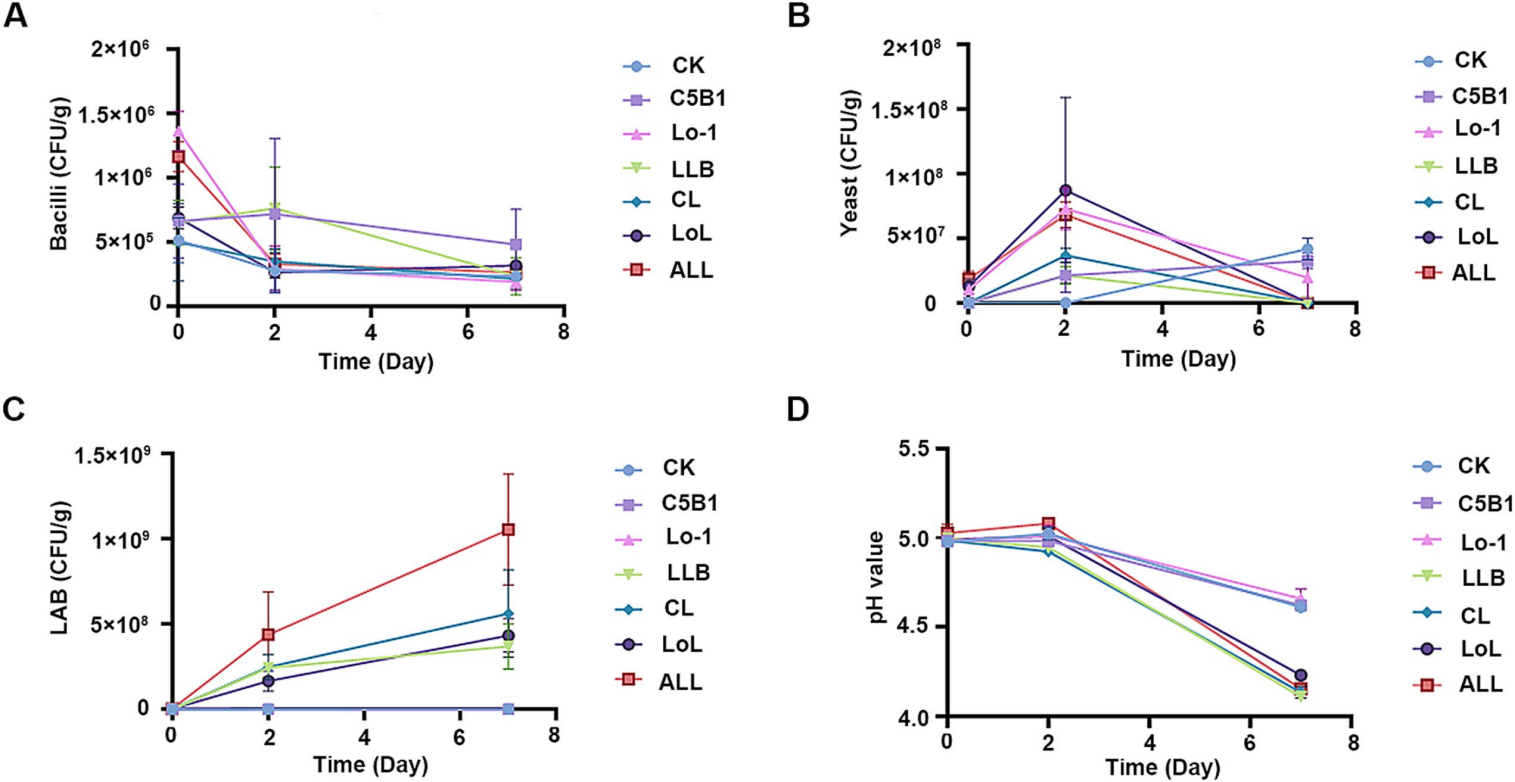
The cultivable microbes and pH values during the fermentation of the medicine residue. Bacilli **(A)**, yeast **(B)**, and lactic acid bacteria (LAB, **C**) were separately counted on the LB agar media, YPD agar media, and MRS agar media. **(D)** The changes of the fermentation pH during the whole process. CK, no exogenous microbes; C5B1, *Bacillus subtilis* C5B1; Lo-1, *Saccharomyces cerevisiae* Lo-1; LLB, *Lactiplantibacillus plantarum* LLB; CL, *B. subtilis* C5B1 and *L. plantarum* LLB; LoL, *S. cerevisiae* Lo-1 and *L. plantarum* LLB; ALL, *B. subtilis* C5B1, *L. plantarum* LLB, and *S. cerevisiae* Lo-1.

To characterize the dynamic changes in the microbial community during the fermentation process of the medicine residues, the populations of cultivable microorganisms were determined (Figure 3). On the initial day of fermentation (day 0), there were no significant differences in the microbial compositions among the groups, including those supplemented with *B. subtilis* C5B1 and *S. cerevisiae* Lo-1 (groups C5B1, Lo-1, and CL). However, after 2 days of aerobic fermentation, the counts of *Bacillus* and yeast in these groups significantly exceeded those in the control group (CK) (Figures 3A,B). This increase might be attributed to the presence of antibacterial substances in the SHL medicine residues, potentially inhibiting the initial growth of the introduced fermenting strains. Following the initial 2 days of aerobic fermentation, *Lpb. plantarum* LLB was introduced, and the mixture was sealed for continued fermentation for an additional 5 days. Subsequently, a significant increase in lactic acid bacteria was observed across all groups post the introduction of the strain LLB (Figure 3C). Notably, the ALL group exhibited the most pronounced increase, reaching 10^9^ CFU/g. The subsequent decline in *Bacillus* and yeast might be associated with oxygen depletion during the sealed fermentation phase.

Throughout the fermentation process, pH changes were closely monitored (Figure 3D). At the end of the initial aerobic fermentation phase, the pH value in all the groups had dropped to approximately 5.0. After the 5-day sealed fermentation, the proliferation of lactic acid bacteria led to elevated lactic acid concentrations, resulting in a further decrease in pH. Notably, the CK, C5B1, and Lo-1 groups showed less pronounced pH reductions, likely due to their reduced lactic acid bacteria populations.

### 3.3 Changes in the flavonoid contents and the antibacterial activities of the fermented medicine residues

**Figure 4:**
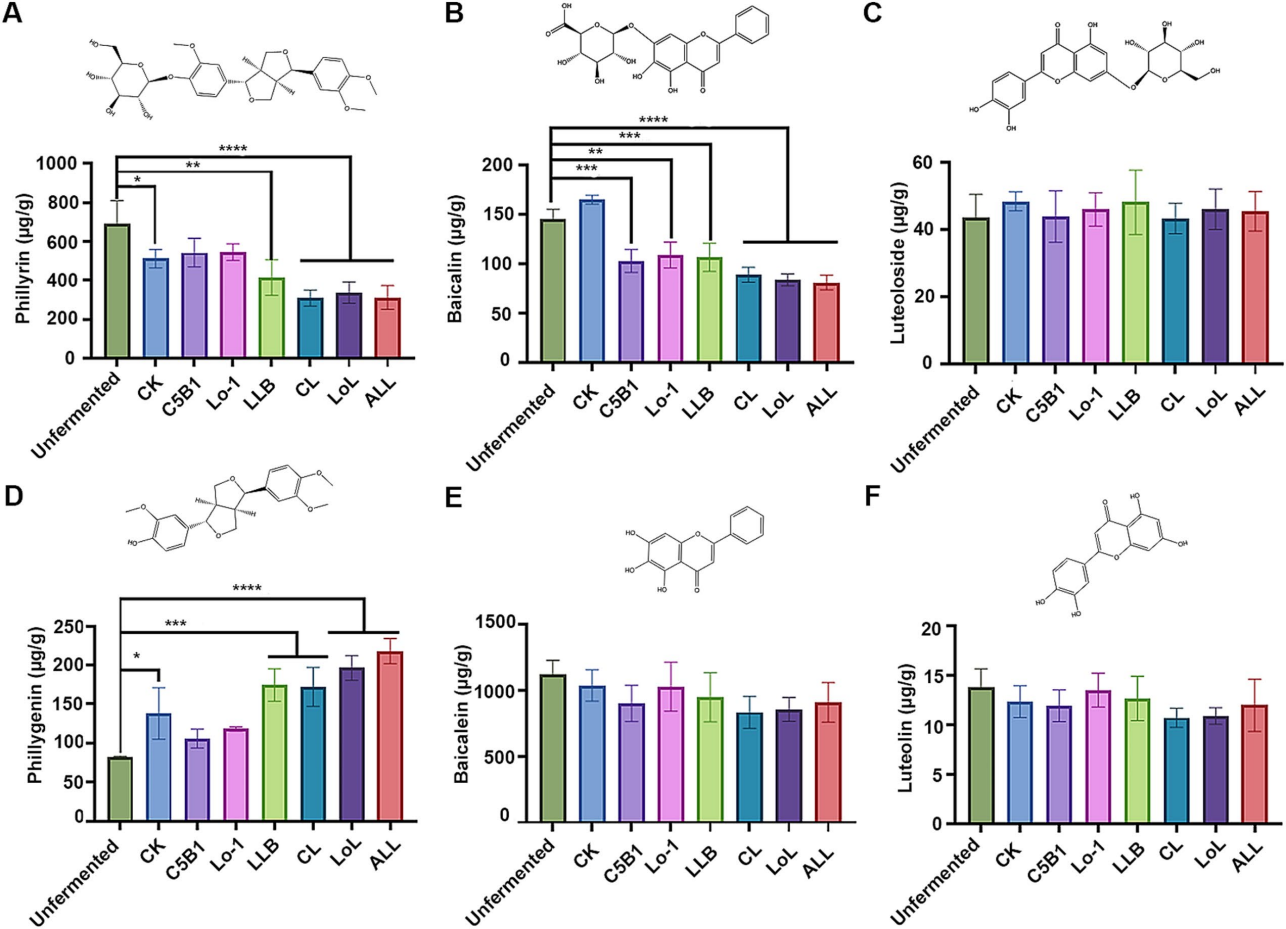
The flavonoid contents of the fermented medicine residues. The contents of phillyrin **(A)** and phillygenin **(D)**, baicalin **(B)** and baicalein **(E)**, as well as luteoloside **(C)** and luteolin **(F)** were determined, respectively. *p < 0.05; **p < 0.01; ***p < 0.001; ****p < 0.0001.

The flavonoid content in the fermented medicinal residues is shown in Figure 4. Compared with the unfermented residue, the phillyrin content decreased significantly in the fermented groups (LLB, CL, LoL, and ALL) with *Lpb. plantarum* LLB, other than the group C5B1and group Lo-1 (Figure 4A). Correspondingly, an obvious increase in the phillygenin content was observed in these same groups (Figure 4D). The result suggested that *Lpb. plantarum* LLB predominantly catalyzed the conversion of phillyrin to phillygenin during the fermentation process. In addition, the baicalin content significantly decreased in all the groups containing the fermenting strains, compared to the unfermented residue (Figure 4B). However, the matching hydrolysis product, baicalein, did not exhibit a notable increase in content (Figure 4E). Given the fermenting strains’ limited capacity to hydrolyze baicalin into baicalein (Figure 1), the result implied that baicalin might have undergone alternative degradation or modification pathways during fermentation. We propose that baicalin (a flavone) may undergo dehydroxylation, a modification previously reported for other flavones (Braune and Blaut, 2016). Dehydroxylation of flavones can be catalyzed by *Escherichia*, which is ubiquitous in environmental microbiota. Alternatively, other microbes in the fermented residues may transform baicalin via O-deglycosylation and C-ring cleavage (Braune and Blaut, 2016), resulting in products distinct from baicalein. Moreover, no significant difference was observed in luteoloside and luteolin contents between the fermented and unfermented groups (Figures 4C,F), suggesting that luteoloside might be recalcitrant to microbial utilization in the fermented residues.

To determine whether the antimicrobial activity of the medicine residue was changed after the fermentation, the inhibitory activities of the fermented residues on pathogens were examined. Antibacterial activity against *Stre. equi* remained unchanged, with no significant difference observed between the fermented and unfermented residue extracts (Supplementary Figure S1A). In contrast, the extract from the unfermented residue demonstrated no inhibitory activity against *E. coli*. The CK, C5B1, and Lo-1 groups also exhibited negligible inhibitory activity against *E. coli*. Conversely, the groups fermented with *Lpb. plantarum* LLB significantly inhibited the growth of *E. coli* (Supplementary Figure S1B). Since the pH in the groups fermented with *Lpb. plantarum* LLB was lower, the enhanced inhibitory activity toward *E. coli* might be attributed to lactic acid produced during the fermentation. Both the unfermented and fermented medicinal residues displayed inhibitory activity against *C. albicans* (Supplementary Figure S1C). However, the activity of the unfermented extract was markedly lower than all the other groups except group C5B1, suggesting that production of active metabolites during the fermentation.

### 3.4 Effects of feeding the fermented medicine residue on the growth performance and meat quality of broiler chickens

Due to the presence of numerous antimicrobial substances, the fermented medicine residue was potential as an alternative to antibiotics. In addition, the presence of other bioactive substances in the fermented residue made it a promising feed additive for application in animal agriculture. In the broiler chicken feeding trail, the ALL group, which exhibited high viable microbial counts and antimicrobial activity, was selected for evaluation as a feed additive. Comparison with the control group (group A), which received a basic diet, revealed no significant differences in body weight, average daily gain, feed-to-gain ratio, or carcass weight for the groups supplemented with the fermented medicine residue (group C-E) or aureomycin (group F) (Table 3). The results indicated that the addition of the fermented medicine residues did not have any adverse effects on the growth performance of the broiler chickens. Moreover, the meat quality assessment, presented in Supplementary Table S1, showed no significant differences in fat content and protein levels between group A and group E. In addition, there were no obvious differences in the pH value or water retention capacity between the two groups. However, while the tenderness and inosine acid content in group E’s meat were higher than those in group A, these differences were not statistically significant. Based on the above results, the integration of the fermented SHL residues into the broilers’ basic diet did not compromise the growth performance or meat quality, suggesting that a portion of the basal diet could be substituted with these residues, which were previously discarded as waste. Compared to the high costs associated with traditional methods of handling medicine residues (such as landfilling or incineration), microbial fermentation is more cost-effective. Moreover, the use of fermented medicine residues as feed additives can reduce feed costs. Consequently, the utilization of fermented medicine residue as a feed additive appeared to be economically viable.

**Table 3:** The growth performances of the broiler chickens.

Indices	Group A	Group B	Group C	Group D	Group E	Group F
Body weight (g)	2,671 ± 229.1	2,580 ± 444.8	2,499 ± 336.3	2,573 ± 178.8	2,680 ± 265.2	2,522 ± 246.1
Average daily gain (g)	24.68 ± 3.272	23.57 ± 6.354	22.67 ± 4.805	22.87 ± 2.554	24.03 ± 3.788	23.80 ± 3.516
Feed-to-gain ratio (g)	3.469 ± 0.458	3.729 ± 1.451	3.742 ± 1.133	3.654 ± 0.424	3.413 ± 0.581	3.619 ± 0.589
Carcass weight (g)	2,367 ± 157.6	2,345 ± 252.7	2,320 ± 78.99	2,253 ± 120.4	2,323 ± 196.3	2,237 ± 178.0

### 3.5 Analysis of antioxidants, cytokines and immunoglobulins in the sera of the fed broiler chickens

**Figure 5:**
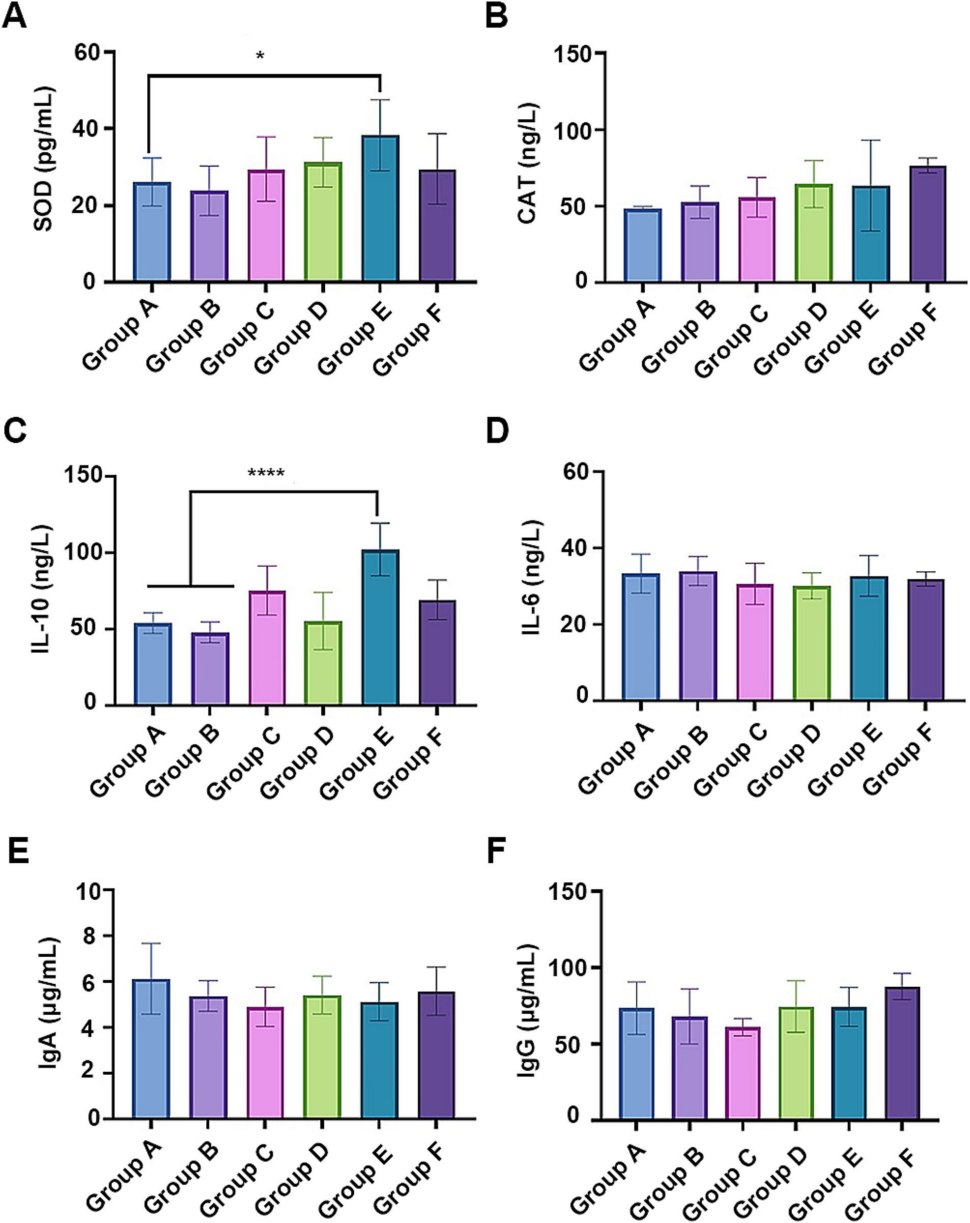
The effects of the fermented medicine residues on the antioxidant system and the immune system of the fed broiler chickens. The serum antioxidant activities were indicated by the contents of superoxide dismutase (SOD, **A**) and catalase (CAT, **B**). The serum cytokines IL-10 **(C)** and IL-6 **(D)**, as well as antibodies IgA **(E)** and IgG **(F)** were examined for evaluating the effects on the immune system. *p < 0.05; ****p < 0.0001.

Given their effective antioxidant properties (Dai et al., 2024; Milicevic, 2024), SHL medicine residue enriched with flavonoids may enhance the antioxidant and immune system of the fed broiler chickens. As expected, the serum superoxide dismutase (SOD) level in broilers fed with a 5% fermented residue diet (group E) significantly increased (*p* < 0.05) (Figure 5A), compared to that on a basal diet (group A). There were no significant differences in the catalase (CAT) or total antioxidant capacity (T-AOC) among the groups (Figure 5B; Supplementary Figure S2A). In addition, compared to the broilers fed with the basic diet or the basic diet with the unfermented medicine residue (group B), the supplementation with 5% fermented residue in the diet significantly elevated IL-10 serum concentration (Figure 5C), while minimally affecting the levels of other cytokines (e.g., IL-6 and IFN-γ) (Figure 5D; Supplementary Figure S2B) and the immunoglobulins (IgA, IgG, and IgM) (Figures 5E,F as well as Supplementary Figure S2C). Given the higher abundance of lactobacilli cells and their active metabolites in Group E compared to the other groups, bacterial cell components such as S-layer proteins (Konstantinov et al., 2008) may contribute to the increased levels of IL-10. These findings suggested that the incorporation of the fermented medicine residue into the diet positively impacted animal health.

### 3.6 Analysis of the intestinal microbial compositions of the fed broiler chickens

**Figure 6:**
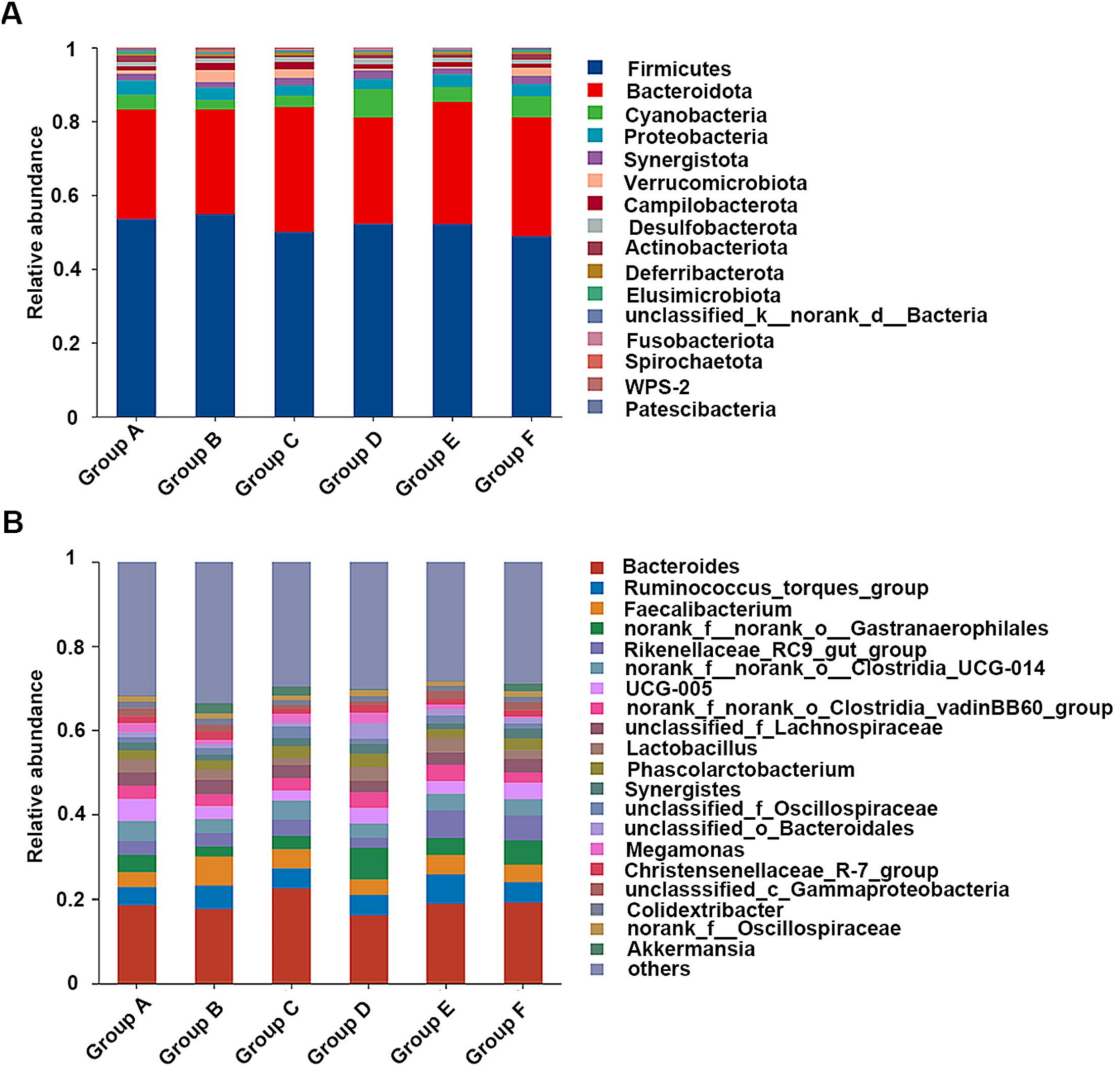
The cecal microbial compositions of the fed broiler chickens at the phylum **(A)** and genus level **(B)**. The diets are basal diets with different additives: blank (group A), unfermented medicine residue (group B), natural fermented medicine residue (group C), fermented medicine residue with mixed microbes (group D and group E), aureomycin (group F).

The intestinal microbial compositions across all the chicken groups were analyzed through high-throughput 16S rRNA gene sequencing. The α-diversity analysis, including Chao, Shannon, and Simpson indices, revealed no significant differences among the groups (Supplementary Table S2). In each group, *Firmicutes* and *Bacteroidota* were the major phyla, constituting approximately 80% of the bacterial community (Figure 6A), aligning with known gut microbiota of broiler chickens (Liu et al., 2023). The relative abundances of *Actinobacteriota*, *Elusimicrobiota*, and *Patescibacteria* were significantly reduced in chickens fed with the naturally fermented medicine residue (group C) compared to those fed with the basal diet (group A) (Supplementary Figure S3A). Conversely, *Spirochaetota* was significantly more abundant in group C than in the groups fed with the fermented residues by the mixed strains (group D and group E) (Supplementary Figure S3B). In addition, group E exhibited significantly lower relative abundances of *Spirochaetota* and *Elusimicrobiota* compared to group A (Supplementary Figure S3C). Similarly, the relative abundance of *Elusimicrobiota* in group E was also lower than in group F, which was treated with the antibiotic aureomycin (Supplementary Figure S3D). Given that the relative abundances of *Elusimicrobiota* were significantly lower in the groups without the medicine residue (Groups A and F) compared to those with the medicine residue (Groups B–E), we hypothesize that antimicrobial substances present in the medicine residues, such as flavonoids and chlorogenic acid (Cushnie and Lamb, 2005; Liu et al., 2023), may be active against *Elusimicrobiota*.

**Figure 7:**
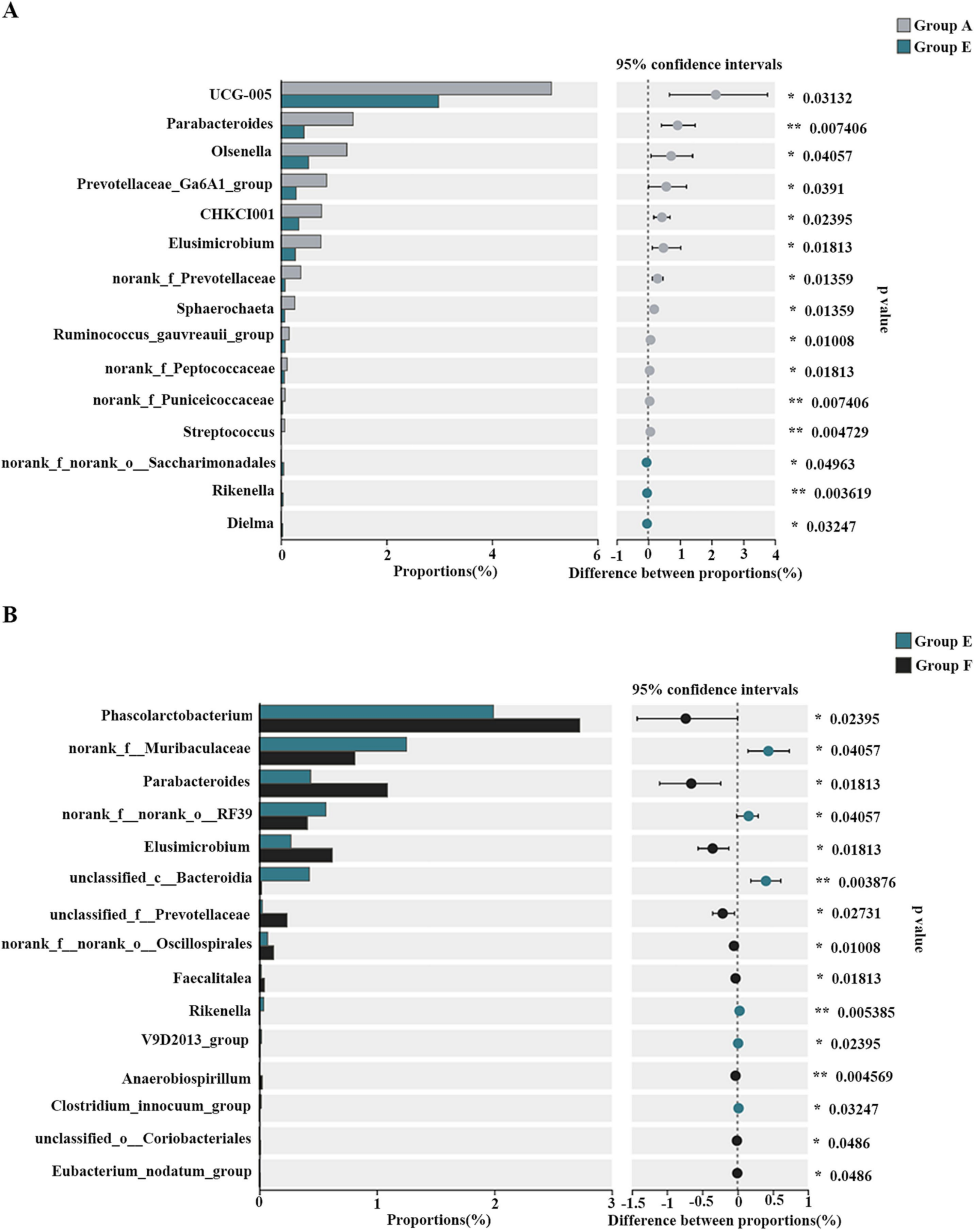
The differential genera of the cecal bacterial communities of the broiler chickens between group A and group E **(A)** as well as group E and group F **(B)**. *p < 0.05; **p < 0.01.

At the genus level, *Bacteroides*, *Ruminococcus*, and *Faecalibacterium* were predominant (Figure 6B). Multiple comparative analyses revealed significant differences in the abundance of certain genera across the groups (Supplementary Figure S4). Specifically, group D and group E exhibited higher levels of unclassified_c*__Bacteroidia* relative to the other groups. Group A showed higher abundances of UCG-005, *Parabacteroides*, *Olsenella*, *Prevotellaceae*_Ga6A1_group, CHKCI001, *Elusimicrobium*, norank_f__*Prevotellaceae*, *Sphaerochaeta*, *Ruminococcus gauvreauii* group, norank_f__*Peptococcaceae*, norank_f__*Puniceicoccaceae*, and *Streptococcus* compare to group E. Meanwhile, other genera, such as norank_f__norank_o__*Saccharimonadales*, *Rikenella*, and *Dielma,* were notably more abundant in group E (Figure 7A). The observed changes in genera differed from those induced by quercetin (Dong et al., 2020), a flavonoid aglycone present in the SHL medicine residue. Additionally, compared to the antibiotic group (group F), the fermented medicine residue group (group E) displayed higher levels of norank_f__*Muribaculaceae*, unclassified_c__*Bacteroidia*, and *Rikenella*. In contrast, group E exhibited reduced levels of *Phascolarctobacterium*, *Parabacteroides*, *Elusimicrobium*, unclassified_f__*Prevotellaceae*, norank_f__norank_o__*Oscillospirales*, *Faecalitalea*, and *Anaerobiospirillum* (Figure 7B). Furthermore, to assess the impact of varying additive amounts of the fermented medicine residue, a pairwise comparison was performed between group D and group E. Group D had significantly higher abundances of *Parabacteroides*, norank_f__norank_o*__Oscillospirales*, and norank_f__*Christensenellaceae*, while group E showed higher levels of norank_f__*Muribaculaceae*, *Subdoligranulum*, and norank_f__norank_o__*Bacteroidales* (Supplementary Figure S5). These significant changes in the gut microbial compositions suggest that SHL medicine residues interact closely with gut microbiota, as previously discussed (An et al., 2019). These interactions may occur through two primary mechanisms: (1) direct modulation of microbial growth via nutritional or antimicrobial effects, and (2) indirect regulation of gut microbiota through host physiological changes mediated by microbial metabolites of the medicine residues. Additional complex interactions among medicine residues, gut microbes, and host cells remain to be elucidated in future studies.

Based on the above results, the fermented SHL medicine residue was positively correlated with the intestinal abundances of unclassified_c__*Bacteroidia*, norank_f__*Muribaculaceae*, and *Rikenella* (Supplementary Figure S4; Figure 7), while inversely associated with those of *Parabacteroides* (Supplementary Figures S4, S5; Figure 7). Notably, *Rikenella*, among the bacteria identified, is able to produce short-chain fatty acids (SCFAs), which can improve the intestinal environment, enhance the intestinal barrier (Li J. et al., 2022), and potentially reduce the incidence of diarrhea in animals. Given that SCFAs are known to regulate the expression of cytokines such as IL-6 and IL-10 (Sugiharto and Ranjitkar, 2019), the increased relative abundance of *Rikenella* in the gut microbiota may contribute to the elevated serum IL-10 levels observed in broiler chickens fed with the fermented medicine residue. Consequently, the fermented SHL medicine residue holds promise as an innovative feed additive, offering benefits for broiler chicken health and contributing to the broader goal of food security.

## 4 Conclusion

In summary, we have isolated and characterized a potential probiotic, *Lpb. plantarum* LLB, from the traditional Chinese medicine residue. The strain efficiently utilized the prebiotic flavonoid glycosides in the medicine residue. We employed microbial transformation to repurpose the medicine residues, previously considered waste, into a novel feed additive, which did not compromise the growth performance or meat quality of broiler chickens. Future work will focus on elucidating the genetic mechanisms underlying the probiotic utilization of flavonoid glycosides. Additionally, the results from the feeding experiment may have potential limitations because of a small sample size and should be further validated through larger-scale trials. Overall, the study provides a valuable and cost-effective approach for managing Chinese medicine residue and advances the application of prebiotics and probiotics in animal agriculture.

## 5 Chicken studies

The feeding experiment was conducted in the experimental farm of Henan Agricultural University, Henan province of China. The experiment protocol was approved by the life ethics review committee of Henan Academy of Sciences (protocol number HNAS.No20231017b006). The sample size was determined using G*Power (Faul et al., 2007), a widely recognized software for power analysis. ANOVA was employed to perform the statistical test. The sample size was calculated based on the following input parameters: effect size *f* = 0.3, significance level α = 0.05, power (1−β) = 0.8, and number of groups = 6. The total sample size was calculated to be 168. Accounting for a 5% attrition rate, the adjusted sample size was determined to be 177. Consequently, 30 chickens were assigned equally to each group.

## Data Availability

The raw sequencing reads were deposited in National Microbiology Data Center with the accession number NMDC10018890.
