# Supplementary material for: Efficient utilization of Shuanghuanglian medicine residues by microbial transformation with flavonoid glycosides-hydrolyzing strains
Source: Front Microbiol. 2025 May 27;16:1553399. doi: 10.3389/fmicb.2025.1553399 (PMC12149203; doi:10.3389/fmicb.2025.1553399)
Supplement: Supplementary file 1 [file Data_Sheet_1.pdf]

Supplementary Materials

Figure S1: The antimicrobial activities of the fermented medicine residues

Figure S2: The serum total antioxidant capacity (T-AOC), IFN-γ, and IgM of the fed broiler chickens

Figure S3: Comparisons of the relative abundances between groups at the phylum level

Figure S4: The relative abundances of significantly different genera among the groups

Figure S5: Comparison of the relative abundances between group D and group E at the genus level

Table S1: The meat qualities of the broiler chickens

Table S2: The α-diversity indices of the intestinal microbiota of the fed broiler chickens

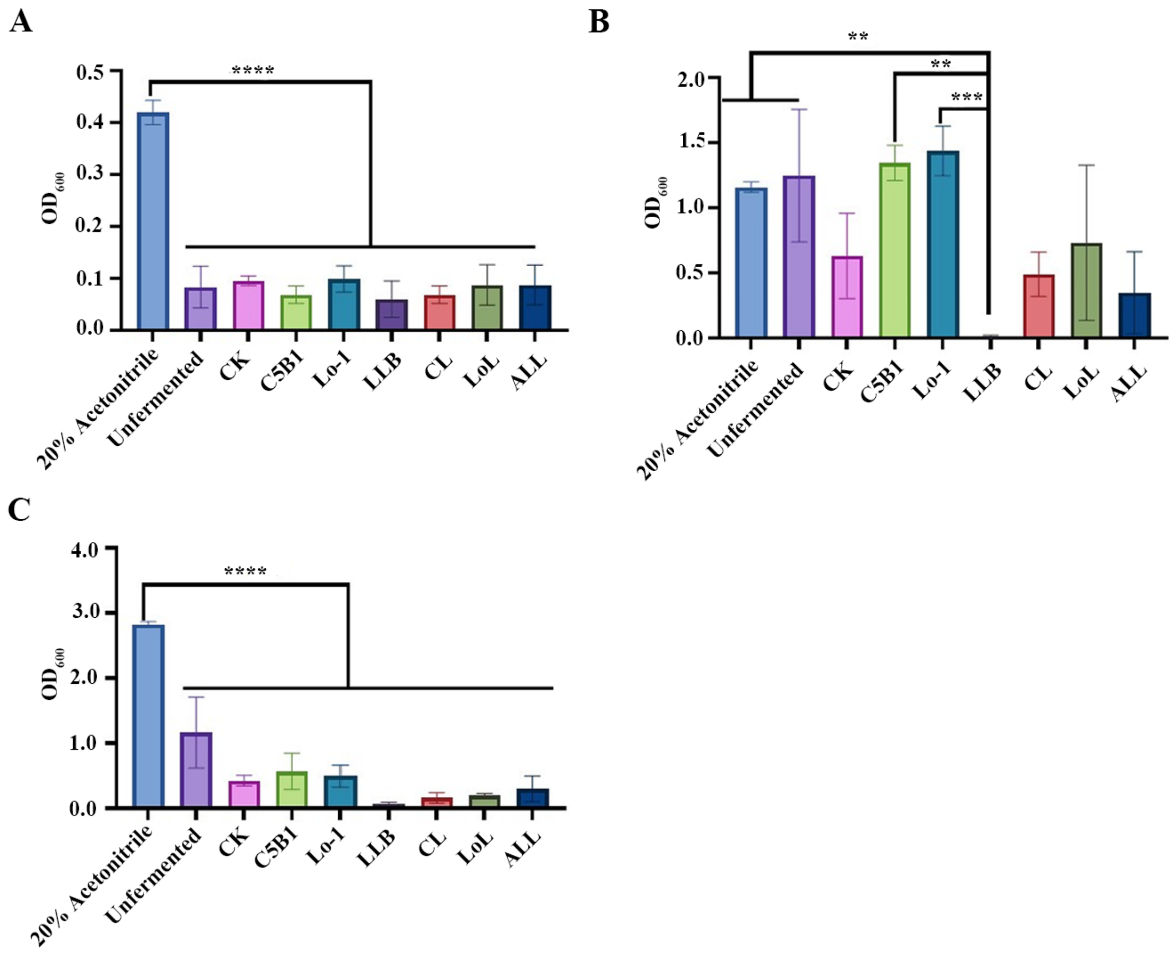

Figure S1. The antimicrobial activities of the fermented medicine residues. *Streptococcus equi* CGMCC 1.10838 (A), *Escherichia coli* DH5α (B), and *Candida albicans* SC5314(C) were used as indicators. $p<0.01$, **; $p<0.001$, ***; $p<0.0001$, ****.

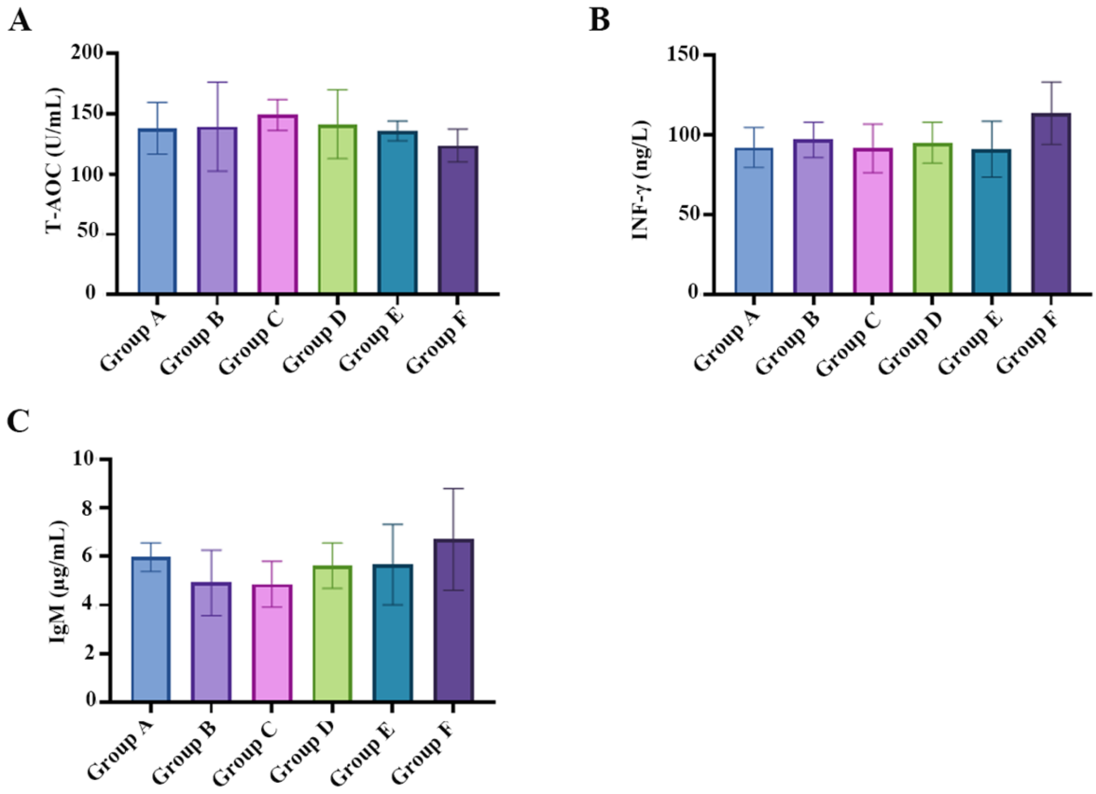

Figure S2. The serum total antioxidant capacity (T-AOC) (A), IFN-γ (B), and IgM (C) of the fed broiler chickens.

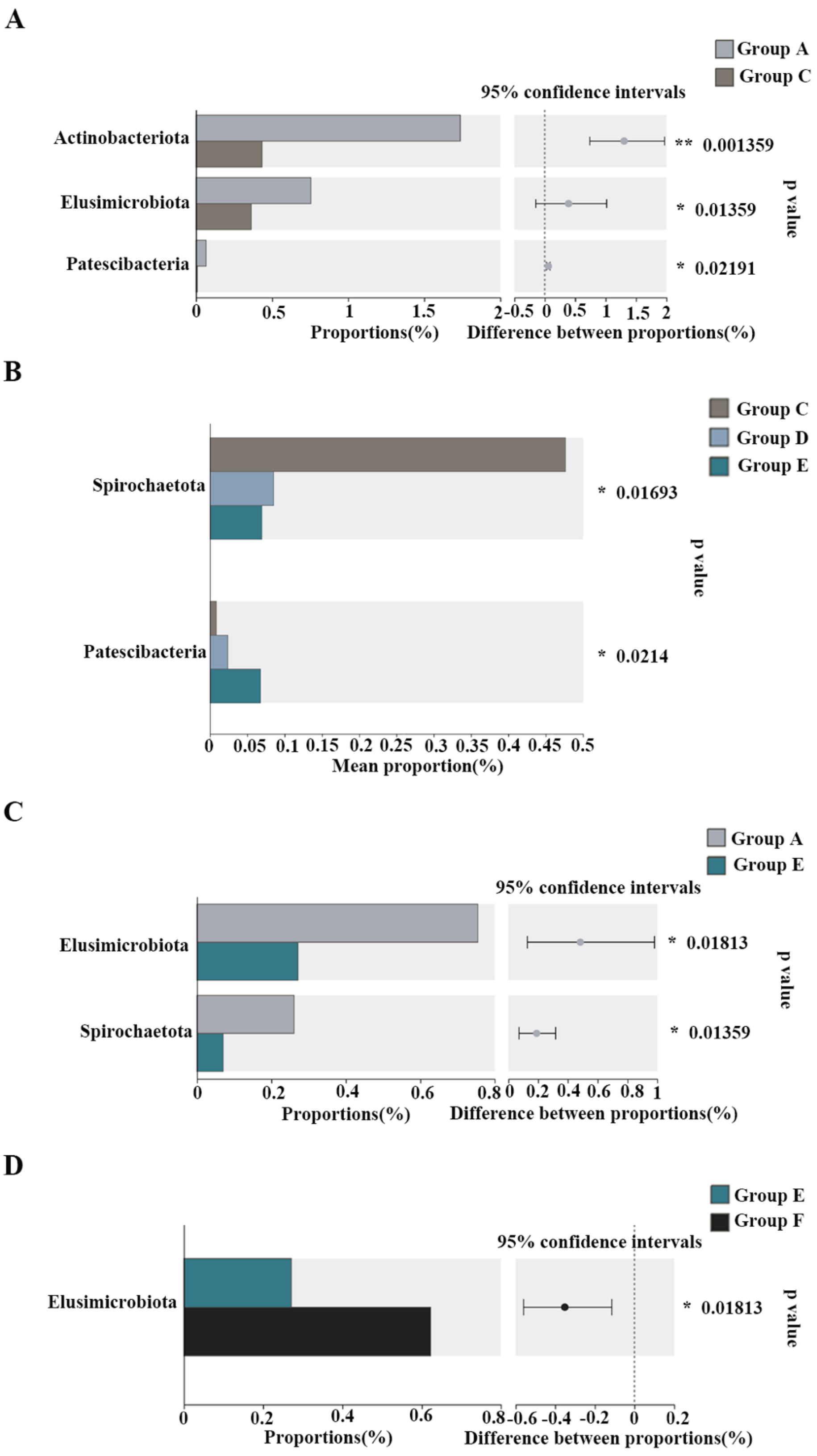

Figure S3. Comparisons of the relative abundances between groups at the phylum level. p<0.05, *; p<0.01, **.

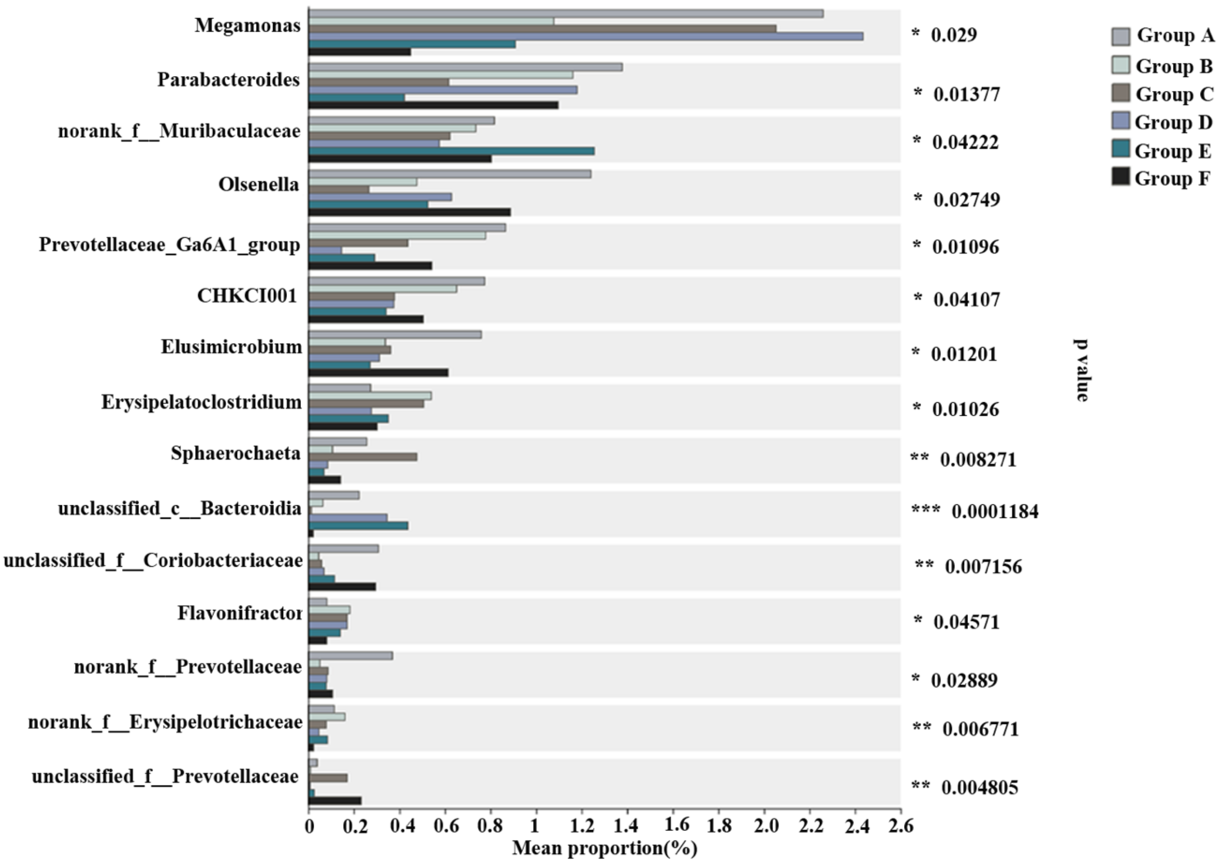

Figure S4. The relative abundances of significantly different genera among the groups. p<0.05, *; p<0.01, **; p<0.001, ***.

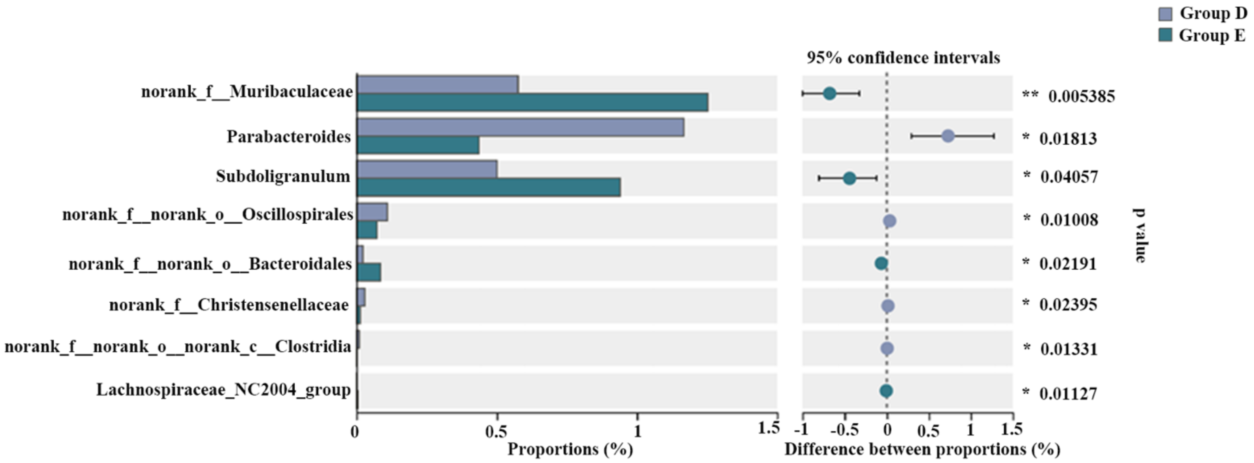

Figure S5. Comparison of the relative abundances between group D and group E at the genus level. $p<0.05$, *; $p<0.01$, **.

Table S1. The meat qualities of the broiler chickens

Indices	Group A	Group E
Fattiness (g/100 g)	1.367±0.116	1.400±0.173
Protein (g/100 g)	23.67±0.116	23.43±0.208
pH value	5.867±0.0231	5.853±0.0379
Water retention capacity (%)	50.53±0.666	50.53±1.266
Tenderness (N)	23.97±1.069	25.23±0.961
Inosine acid (mg/g)	1.640±0.191	1.830±0.209

Table S2. The α-diversity indices of the six groups

Estimators	Group A	Group B	Group C	Group D	Group E	Group F	P value
Ace	2081 ± 600.0	1942 ± 176.6	1955 ± 259.8	1890 ± 199.1	1994 ± 392.9	2120 ± 201.8	0.2139
Chao	1944 ± 537.5	1813 ± 152.2	1831 ± 228.1	1791 ± 144.0	1862 ± 335.6	1981 ± 167.6	0.1513
Coverage	0.9849 ± 0.00484	0.9855 ± 0.00188	0.9853 ± 0.00223	0.9859 ± 0.00165	0.9852 ± 0.00343	0.9838 ± 0.00161	0.2268
Shannon	5.303 ± 0.4419	5.144 ± 0.2373	5.057 ± 0.4335	5.180 ± 0.1490	5.108 ± 0.2618	5.186 ± 0.1655	0.5788
Simpson	0.01974 ± 0.014850	0.02115 ± 0.008007	0.03047 ± 0.031440	0.01970 ± 0.006923	0.02386 ± 0.008775	0.02018 ± 0.005515	0.5441
Sobs	1632 ± 468.6	1472 ± 119.1	1478 ± 175.2	1440 ± 158.8	1532 ± 289.0	1603 ± 181.4	0.2137
